# Inequitable Access to Transplants: Adults With Impaired Decision-Making Capacity

**DOI:** 10.3389/ti.2022.10084

**Published:** 2022-03-18

**Authors:** Rebecca L. Thom, Anne Dalle-Ave, Eline M. Bunnik, Tanja Krones, Kristof Van Assche, Alex Ruck Keene, Antonia J. Cronin

**Affiliations:** ^1^ Kings College Hospital London, London, United Kingdom; ^2^ Ethics Unit, Institute of Humanities in Medicine, University Hospital of Lausanne, London, United Kingdom; ^3^ Department of Medical Ethics, Philosophy and History of Medicine, Erasmus Medical Centre, Rotterdam, Netherlands; ^4^ Department of Clinical Ethics, University Hospital Zurich, Zurich, Switzerland; ^5^ Institute of Biomedical Ethics and History of Medicine, University of Zurich, Zurich, Switzerland; ^6^ University of Antwerp, Antwerp, Belgium; ^7^ 39 Essex Chambers, London, United Kingdom; ^8^ King’s College London, London, United Kingdom; ^9^ Guy’s and St. Thomas’ NHS Trust and King’s College London, London, United Kingdom

**Keywords:** transplantation, ethics, capacity, law and policy, equitable access

## Abstract

Inequitable access to deceased donor organs for transplantation has received considerable scrutiny in recent years. Emerging evidence suggests patients with impaired decision-making capacity (IDC) face inequitable access to transplantation. The “Ethical and Legal Issues” working group of the European Society of Transplantation undertook an expert consensus process. Literature relating to transplantation in patients with IDC was examined and collated to investigate whether IDC is associated with inferior transplant outcomes and the legitimacy of this healthcare inequality was examined. Even though the available evidence of inferior transplant outcomes in these patients is limited, the working group concluded that access to transplantation in patients with IDC may be inequitable. Consequently, we argue that IDC should not in and of itself be considered as a barrier to either registration on the transplant waiting list or allocation of an organ. Strategies for non-discrimination should focus on ensuring eligibility is based upon sound evidence and outcomes without reference to non-medical criteria. Recommendations to support policy makers and healthcare providers to reduce unintended inequity and inadvertent discrimination are set out. We call upon transplant centres and national bodies to include data on decision-making capacity in routine reporting schedules in order to improve the evidence base upon which organ policy decisions are made going forward.

## Introduction

Issues of scarce resource allocation and inequitable access to medical treatment have long-since been the doctor’s dilemma. Deceased donor organs for transplants are a scarce resource, and it is widely agreed that equitable access to transplantation must be prioritised. In recent years transplant professionals and advocacy groups have highlighted how those who may have impaired legal decision making capacity (IDC) have historically faced inequitable access to transplant waiting lists and organ allocation (1–3). This has led to multiple United States jurisdictions instituting specific legislation, however such changes are yet to be seen in Europe (1).

Those who may have IDC include patients with 1) intellectual disability, 2) a mental health condition, including for example disorders affecting reasoning such as psychosis, 3) cognitive impairment that may be due to neurological disease or a single acquired deficit (e.g., stroke or head injury) and finally 4) disorders or consciousness such as persistent vegetative or minimally conscious states. Cognitive impairment is of particular importance as up to 70% of patients aged over 55 receiving dialysis have moderate to severe cognitive impairment (4) and there is emerging evidence which suggests such patients have a lower likelihood of being listed for transplantation (5).

In this paper we interrogate the relationship between 1) apparent lack of mental capacity to make relevant decisions and 2) equitable access to deceased donor organ transplantation. We seek to explain why lacking the mental capacity to consent to transplant should not itself per se be a barrier to access to and allocation of an organ for transplant. We do this with reference to four key transplant outcome measures and specifically interrogate whether, and if so to what extent, the concerns raised by these four key transplant outcome measures are supported by published empirical evidence. We highlight ethical considerations and legal issues, and, finally, set out recommendations and guidelines for clinicians and policy makers to help overcome perceived barriers and avoid unintentional discrimination.

## Materials and Methods

The “Ethical and Legal Issues” working group of the European Society of Organ Transplantation undertook an expert consensus process between October 2020 and March 2021. This took the form of extensive online discussions between clinical transplant, ethics, and legal experts. Discussions were informed by a review of the published literature relating to transplantation in persons with IDC.

For the purpose of this paper relevant literature was identified by a search of MEDLINE accessed through PubMed. Search terms used were (organ transplantation) AND (mental incapacity OR intellectual disability) between September 2010 and September 2020. We included peer reviewed publications from scholarly journals. Our key purpose was to identify whether strong evidence existed to support the view that transplant outcomes are inferior in persons with IDC.

Our search generated 66 papers. The titles and abstracts of all English language papers were screened. 16 papers relevant papers were identified. One paper was excluded as it was a case study. Seven papers were primary research- six retrospective cohort studies and one online survey. The remainder were literature reviews, ethical analyses or editorials. Further sources were identified through cited materials. In addition, primary and secondary legal sources from LexisNexis and Westlaw databases and public policy documents were analysed.

## Transplant Outcome Measures and Inequitable Access to Transplantation

Four key transplant outcome measures emerge in the literature as relevant clinical concerns and to varying degrees cut across all the groups we have identified as at risk of lacking the mental capacity to make relevant decisions as regards to medical treatment and transplantation. These are 1) medication adherence, 2) graft outcome, 3) patient outcome and 4) quality-of-life (QoL). While medication adherence is not itself a transplant outcome measure, we observe that medication non-adherence is assumed to have a causal effect on transplant outcomes. As post-transplant medication non-adherence is taken to negatively impact organ and patient survival and quality of life, the *prognosis* of non-adherence is mentioned in the literature as a reason not to list a patient or not to allocate an organ.

We assessed whether, and, if so, to what extent, the concerns raised by these four inter-related key transplant outcome measures are supported or actively refuted by the published empirical evidence. We included outcome data relating to living donor transplantation because limited evidence was available on deceased donor transplant outcomes in persons with IDC. A summary of this empirical assessment is set out in table one (Table 1) and is followed by an ethical and legal analysis of the concerns raised by each transplant outcome measure and by their assumed causal dependency.

**TABLE 1:** Summary of empirical evidence relating key transplant outcome measures to each group with potentially impaired decision making capacity.

Group with potentially impaired DECISION-MAKING capacity	Key transplant outcome measures			
	Adherence with medical therapy	Graft outcome	Patient outcome	Quality of life
Intellectual disability	Cohort studies suggesting adherence is comparable. OCEBM^a^ level 3 (1, 17)	Multiple cohort studies suggesting graft outcomes are comparable. OCEBM level 3 (1, 13–19, 33)	Multiple cohort studies suggesting non-graft outcomes are comparable. OCEBM level 3 (1, 13–19, 33)	Evidence is that in general quality of life is improved by transplantation (25)
				OCEBM level 1
				Small number of cohort studies showing QOL benefit in this group. OCEBM level 3 (26, 33)
Severe mental health conditions	Evidence of increased non-adherence in those with depression (7) OCEBM level 3 but not in other conditions in particularly in those with psychosis/mania (8, 9) OCEBM Level 3	Evidence of poorer outcomes in those with depression (24) OCEBM level 1. Otherwise conflicting evidence from cohort studies of other psychological conditions OCEBM Level 3 (2, 8, 9, 22)	Evidence of poorer outcomes in those with depression (24) OCEBM level 1	Evidence is that in general quality of life is improved by transplantation (25)
			Otherwise conflicting evidence from cohort studies of other psychological conditions OCEBM Level 3 (2, 8, 9, 22)	OCEBM level 1
Cognitive impairment	Evidence from cohort studies of reduced adherence in older age groups of transplant recipients	Cohort studies indicate worse outcomes (23)	Cohort studies indicate worse outcomes (23)	Cohort study evidence that QoL benefit is consistent in over 65s (those most at risk of cognitive impairment on dialysis)(28)
	OCEBM level 3 (11, 12)	OCEBM level 3	OCEBM level 3	OCEBM level 3
Permanent disorders of consciousness	No concern as adherence would be assured by caregiver	No evidence available	No evidence available	Theoretical reason to believe QoL outcomes would be significantly different from the general population of transplant recipients

a Oxford Centre for Evidence Based Medicine 2011 levels of evidence are included to indicate the degree of certainty with which the authors make these assertions.

This table has drawn on evidence relating to intellectual disability from the paediatric literature. However in this paper we do not consider children as a discrete category, as they are treated differently where they are considered too young to have the legal capacity to make the relevant decisions, whether or not they have any intellectual disability or mental disorder.

In the empirical and theoretical literature found to date disorders of consciousness and their implications for potential transplant recipients have not received attention. This lack of empirical evidence has led us to exclude them from our further discussion, although their position would benefit from further theoretical analysis as they seem to be a group who are subject to distinct concerns.

### Medication Adherence

Non-adherence to prescribed medication is common, transplantation is no exception. The estimated prevalence of non-adherence in transplant recipients is between 36 and 55% (6). There are multiple factors which have been shown to be associated with non-adherence, including “youth (<50 years old), male, low social support, unemployment, low education, >3 months post graft, living donor, >6 comorbidities, >5 drugs/day, >2 intakes/day, negative beliefs, negative behaviour, depression and anxiety (7)”- however, many of these factors may be equally present in patients who have decisional capacity as in those who lack it.

Non-adherence is frequently linked to those with mental health disorders (2). However, in a study of 955 transplant recipients it was found that those with a pre-existing mental health diagnosis and those with pre-transplant non-adherence were not necessarily groups which overlapped (8). Studies looking specifically at adherence in severe mental health disorders which may result in IDC (e.g., psychosis) are scarce. However Molnar used percentage of days covered by immune suppression prescriptions for a cohort of 442 post-transplant patients with a history of psychosis and mania and found that these did not differ significantly between those with a psychiatric history and those without (9).

In contrast it could be argued that those with intellectual disability may already have strong social support networks and committed carers which act as protective factors against non-adherence (1, 10). Samelson-Jones in a case review of five adults with intellectual disability who received cardiac transplants found only one instance of significant non-adherence which was primarily due to a deterioration in the ability of the caregiver rather than the patient (10).

Finally, it is widely acknowledged that in the general population those with advanced age and co-morbidity face specific barriers to adherence. Polypharmacy, visual loss and cognitive impairment may all contribute to difficulty adhering with complex medication regimes. One study which attempted to assess if these general concerns were replicated in the transplant population showed non-adherence to be alarmingly high in older transplant recipients affecting 86% (11). With another showing that age >60 was found to be significantly associated with worse adherence (12).

The limited evidence available is inconclusive with regards to whether adherence in persons with IDC is reduced when compared to the general population. It is therefore not possible to assert that IDC can legitimately be used as a surrogate marker for post-transplant non-adherence. Concerns related to post-transplant medication non-adherence may be alleviated when committed caregivers and social support networks are available.

### Graft and Patient Outcomes

Cohort studies have shown that patients with intellectual disability receiving a variety of solid organ transplants have equal survival to those without (1, 13–20). A literature review of transplant outcomes in those with intellectual disability found 18 published studies with a mixture of solid organ transplants included, mostly but not exclusively in paediatric recipients (1). The largest cohorts are found in kidney transplant recipients where 5-year graft survival ranged from 75 to 100% (1) and when compared to matched populations without intellectual disability there is no difference in acute rejection or graft survival (13).

Meta-analysis have shown depression to be associated with increased graft loss and all-cause mortality RR1.65 (CI:1.21–2.26) (21) although a causative factor is not considered and a large retrospective cohort study of 4582 patients in Ontario has shown a hazard ratio (HR) = 1.494 [95% confidence interval (CI) = 1.168–1.913] of post-transplant death in patients with a diagnosis of “psychological conditions” which was independent of age (22). However, this represents a very heterogenous group. In contrast cohort studies of patients with psychosis or mania do not reveal an association with increased rejection or graft loss (8, 9) although there is likely to be selection bias as those transplanted were likely stable prior to transplantation.

Cognitively impaired recipients in a retrospective study of 864 patients at two centres in North America showed that there was a substantially higher all cause graft loss than in those without impairment in living donor recipients- aHR 5.40 (CI 1.78–16.34, *p* < 0.01) and in deceased donor recipients with severe cognitive impairment aHR 2.92 (CI1.13–7.50, *p* = 0.03) but no statistically significant difference in those with any stage of cognitive impairment (23).

### Quality of Life (QoL)

There is a wealth of evidence supporting the assertion than kidney patients’ QoL is greatly improved by transplantation, particularly when compared to remaining on dialysis (25). This is the principal reason transplantation is considered to be the gold standard treatment of kidney failure. However, there remains considerable debate over the best measures to judge QoL. For example, a major criticism of the objective Quality Adjusted Life Year (QALY) measure, which gives weight to quantity and utility of life as well as quality, is that it is inherently biased against those with limited life expectancy and that the “Quality” factor is often not measured by self-assessment but by third-party assessment although it is widely recognized that QoL is a subjective rather than an objective dimension.

Chen et al. directly address this with regard to patients with intellectual disabilities and argue that there is “bias, subjectivity and stigma frequently associated with clinicians QoL assessments of patients with intellectual disability [which must] not be used to categorically exclude patients from lifesaving and life-enhancing surgery” (1). They go on to cite evidence that perceived QoL of recipients with intellectual disability and QoL of the principle carer improved post transplantation (26), showing that those with intellectual disability also benefit from transplantation. When considering psychological disorders while psychiatric comorbidity and particularly depression remain common in patients post transplant (27) it does not follow that patients with these diagnoses would be excluded from the benefit to QoL offered by transplantation. Similar criticisms of ableism may be levelled at clinician attitudes towards those with advanced age and cognitive impairment even though again limited evidence would show that QoL improvements from transplantation are consistent even in older age groups (28).

From available evidence on these four interrelated outcomes, one can conclude that there is very limited evidence on non-adherence of persons with IDC, only very weak evidence of worse outcomes of renal transplants with regards to graft and patient survival and QoL in persons with cognitive impairments and/or persons suffering from depression, but not in patients with intellectual disabilities and other psychological conditions.

## Ethical Issues

Clinical decision-making regarding access to or allocation of deceased donor organs for transplant is constrained by scarcity, and so prompts considerations of justice. Justice implies that equals should be treated equally: when patients are similar in medically relevant respects, they ought to be treated equally, as all persons are considered as having the same right to life and health. However, reasonable persons may commit to different ethical theories on what equal treatment entails. Consequently, there is no consensus on the principles of fair allocation of scarce healthcare resources (29).

In living donation, by contrast, the issue of fair allocation does not usually arise, as the recipient brings his or her own donor and does not lay claim to a public pool of scarce organs. That is not to say that there are no ethical concerns regarding equal access in living donation. For instance, access to living donors may not be equally distributed among patients with impaired decision-making capacity. Also, our literature reveals data suggesting significantly inferior outcomes in living donor kidney transplantation in cognitively impaired patients. These concerns merit further investigation, but are beyond the scope of this manuscript.

The most prominent ethical theories of justice are utilitarian and egalitarian. Utilitarian principles aim to maximise the aggregated benefits produced by scarce resources, while egalitarian principles strive for equity or equal opportunity, regardless of aggregated outcomes, and/or for giving priority to the worst-off. These principles for allocation almost always stand in tension with each other, as giving priority to the worst-off often reduces overall utility, and vice versa.

Applying either theory, patients with IDC should be assessed and might even be prioritized, to ensure equal opportunity to a life-saving treatment. It seems reasonable to assume that for all potential recipients, regardless of decisional capacity, transplantation would offer significant QoL benefits, and that assumptions to the contrary may be subject to negative bias. Even from a utilitarian perspective, differentiated treatment of patients with and without relevant decision-making capacities is warranted only when there are (measurable) differences in transplant outcomes between the two groups. The evidence base would have to be as solid and the estimated risk of shorter survival or QoL would have to be as low as in other patients who are currently not being assessed for organ transplant, for example patients with significant cardiovascular or neoplastic disease. Given the current state of knowledge, we conclude that there is no sound ethical justification not to list patients with IDC who (presumably) want to be listed.

Further research is recommended to confirm whether graft or patient outcomes are inferior in patients with impaired decision-making capacity. Evidence on transplant outcomes is needed to guide decision-making about listing for transplantation. However, as long as there is no evidence to conclude that transplant outcomes measures are (much) lower in persons with impaired decision-making capacity, there is no medical or ethical reason to exclude these patients from organ transplantation.

## Legal Issues

The critical legal issue is how to secure individuals with IDC effective legal protection against discrimination on the basis of disability, as this is contrary to the United Nations Convention on the Rights of Persons with Disabilities (CRPD), the European Convention on Human Rights, and many national Constitutions. The CRPD explicitly imposes an obligation upon States party to it to prevent discriminatory denial of health care or health services on the basis of disability (Article 25(f)), as part of those States’ recognition that persons with disabilities have the right to the enjoyment of the highest attainable standard of health without discrimination on the basis of disability. Whilst the European Convention on Human Rights does not include an express right to health, it enshrines in Article 14 the right not to be discriminated against (including on the basis of disability) in the enjoyment of rights under the Convention, including the right to life (Article 2) and the right to physical integrity (Article 8). These obligations are mirrored in non-discrimination provisions enshrined in many national Constitutions. In some of these Constitutions, such as the German Constitution (Article 3 (3)), discrimination on the ground of disability is explicitly prohibited. In short, making eligibility for organ transplantation contingent upon the person’s decision-making capacity would amount to unjustified differential treatment on the basis of intellectual disability, which would be in violation of non-discrimination obligations under human rights and constitutional law. However, existing international guidelines on transplantation do not expressly address the potential for discrimination upon the basis of disability (30–32).

Our concern is that when making decisions about listing or allocation, clinicians might look to the absence of decision-making capacity rather than to the possible relevant medical implications of that incapacity, and, no doubt inadvertently, risk discrimination. That a person may have an intellectual disability means that they may not ask to be put forward for transplantation, but it says nothing about whether they should medically qualify for it.

Therefore, we suggest that transplant wait listing and allocation decisions should take into account decisional incapacity only to the extent that it influences relevant medical criteria, such as the state of that person’s health or the outcome of the transplantation. Also, clinicians should proceed on the basis that a patient without the relevant decisional capacity would wish to be considered for a transplant unless there is good reason to believe to the contrary. This means that focus is then placed upon whether there is a *medical* reason for not putting the person forward.

Further, securing the rights of those with disabilities requires tailoring of care plans, and identifying strategies to support their adherence. Ironically, many of those who lack decisional capacity are in fact in situations where adherence can be maximised, if not guaranteed: for instance those with profound impairments needing continued and intensive care. The most creative of these strategies may be required where a person has fluctuating capacity, for instance as a result of a mental health condition. In some jurisdictions, these strategies could include the approval by court of a care plan aimed at optimising outcome.

Crucially, adopting such strategies (and our recommendations below) will not mean that individuals with impaired decision-making capacity will automatically jump the allocation queue; rather, it means that they are given their proper place in the queue.

## Key Recommendations

The purpose of these recommendations is to promote equitable access to transplantation and ensure that patients without the relevant decisional capacity will be considered for transplantation.

1. That the person does not have the mental capacity to make relevant decisions (“the relevant decisional capacity”) should not in and of itself be an absolute or relative contraindication to transplantation
2. There should be a general assumption that patients without the relevant decisional capacity should have equitable access to organs for transplant and would want to be considered for a transplant unless there is proper reason to believe to the contrary.
3. Decision-making regarding access to transplantation for patients with impaired decisional capacity should as far as possible include the potential recipient, their families and carers. Such decision-making should specifically include 1) identification of the wishes and feelings of the patient towards transplantation; and 2) where it is understood that the patient would wish access to transplantation, drawing up a care plan which would maximise the chances of a successful transplant outcome.
4. When it is being determined that a person without the relevant decisional capacity is not eligible for transplant this must be based on sound medical reasons and evidence. It should not be on the assumption that the lack of capacity in and of itself would affect transplant outcome measures.
5. When a patient without the relevant decisional capacity has been judged not to be suitable for a transplant it is the clinician’s responsibility to inform them and their family/carers honestly and transparently about the basis upon which the decision was made.
6. In order to overcome perceived barriers and avoid unintentional discrimination, transplanting centres and national bodies should include data on decision-making capacity in their routine transplant reporting schedule in order to improve the evidence base upon which organ policy decisions are made going forward, and develop a suitable operational framework that facilitates transplantation in persons with impaired decision-making capacity.
7. International guidelines on transplantation should include, in their provisions on prohibiting discrimination in organ allocation, an explicit reference to discrimination based on disability.

### Conclusion

This paper arose out of a concern on the part of the expert group as to the place of decisional capacity in considerations of access to and allocation of organs for transplants, and, in particular, a concern that such capacity–a cornerstone of autonomy–could inadvertently give rise to unintended discrimination upon the basis of disability. In the paper, we have outlined the ways in which the evidence does not support some of the assumptions which on occasion appear to have underpinned thinking in this area, examined the ethical arguments, and framed matters by reference to international and regional human rights instruments.

We recognise that this paper is just a first start in identifying the problem. We tentatively suggest that our recommendations may assist both in delineating it fully and resolving it. A systematic review to interrogate the issues we have raised further alongside a programme of research investigating transplant outcomes would be useful. Finally, while our focus in this paper has been access to deceased donor organs for transplantation we would like to acknowledge that issues related to living donor transplantation also require attention. In particular, determining whether, and if so to what extent, patients with cognitive impairment have inferior transplant outcomes should be a priority and could help guide clinicians in identifying individuals who may not be suitable for transplantation.

## Abbreviations

IDC, Impaired decision-making capacity; EBPG, European Best Practice Group; QoL, Quality of Life; QALY, Quality Adjusted Life Year; aHR, adjusted hazard Ratio; CI, Confidence Interval; OCEBM, Oxford Centre for Evidence based medicine.
